# Assessing usability of electronic patient-reported outcome measures in older people with and without a rare dermatologic disorder

**DOI:** 10.1186/s41687-024-00821-w

**Published:** 2024-12-18

**Authors:** Calvin N. Ho, Anna Kündig, Lila Bahadori, Katy Roat, Rachel Bruce, Caroline P. Goswami, Kimberly Kelly, Thomas Moll

**Affiliations:** 1 Patient Centered Science, BioPharmaceuticals Medical, AstraZeneca, Gaithersburg, MD USA; 2 Patient Centered Science, BioPharmaceuticals Medical, AstraZeneca, Gothenburg, Sweden; 3 Late-stage Respiratory and Immunology, BioPharmaceuticals R&D, AstraZeneca, Gaithersburg, MD USA; 4 Patient Centered Solutions, IQVIA, New York, NY USA; 5 eCOA Science, Clario, Geneva, Switzerland

**Keywords:** Bullous pemphigoid, Data collection methods, Elderly, eCOA, Electronic clinical outcome assessment, Older adults, Patient-reported outcomes, Electronic patient-reported outcome measure, ePROM, User experience

## Abstract

**Background:**

Robust and well-defined data collection is important when using electronic patient-reported outcome measures (ePROMs) in clinical studies. Questions have been raised as to whether older age may be a barrier to data collection due to patients’ unfamiliarity with electronic devices. Older adults may also have underlying health conditions that affect their ability to fill out patient-reported outcome measures (PROMs) on electronic devices. The aim of this observational, qualitative research study was to evaluate the usability of electronic PROMs (ePROMs) on a tablet and smartphone in older participants with and without bullous pemphigoid (BP).

**Methods:**

Older people with and without BP were recruited in the US and France. They participated in 60-min in-person interviews, with moderators observing their completion of various tasks, including ePROMs, using a tablet and smartphone. Participants were scored on ease of task completion using a scale from 1 to 5.

**Results:**

A total of 12 participants were recruited (≥65 years old; six each with and without BP [all participants without BP were ≥75 years old]). Most participants (83%) could easily and confidently perform most assigned tasks on both the tablet and smartphone. Although select tasks required assistance, all participants were eventually able to complete all tasks. Overall, ePROM usability did not correlate with age, sex, country, or disease state. Feedback on the general usability of both electronic devices was largely positive, and most participants (n = 11; 92%) were willing to use them. Participants were generally pleased with the training modules offered on both devices, describing the training as sufficient, straightforward, and helpful. In total, 25 usability issues were identified, which fell into three categories: incomplete instructions, unclear language, and insufficient technical/visual design. Participants provided feedback on how to enhance device usability.

**Conclusions:**

The results suggest that older people can confidently use a tablet or smartphone for ePROM completion, particularly with appropriate training. ePROMs should be designed with the needs of the target patient population in mind. These results can be leveraged to improve clinical data recording, optimize device usability, and enhance the user experience for older people and those with functional or physical limitations.

**Graphical abstract:**

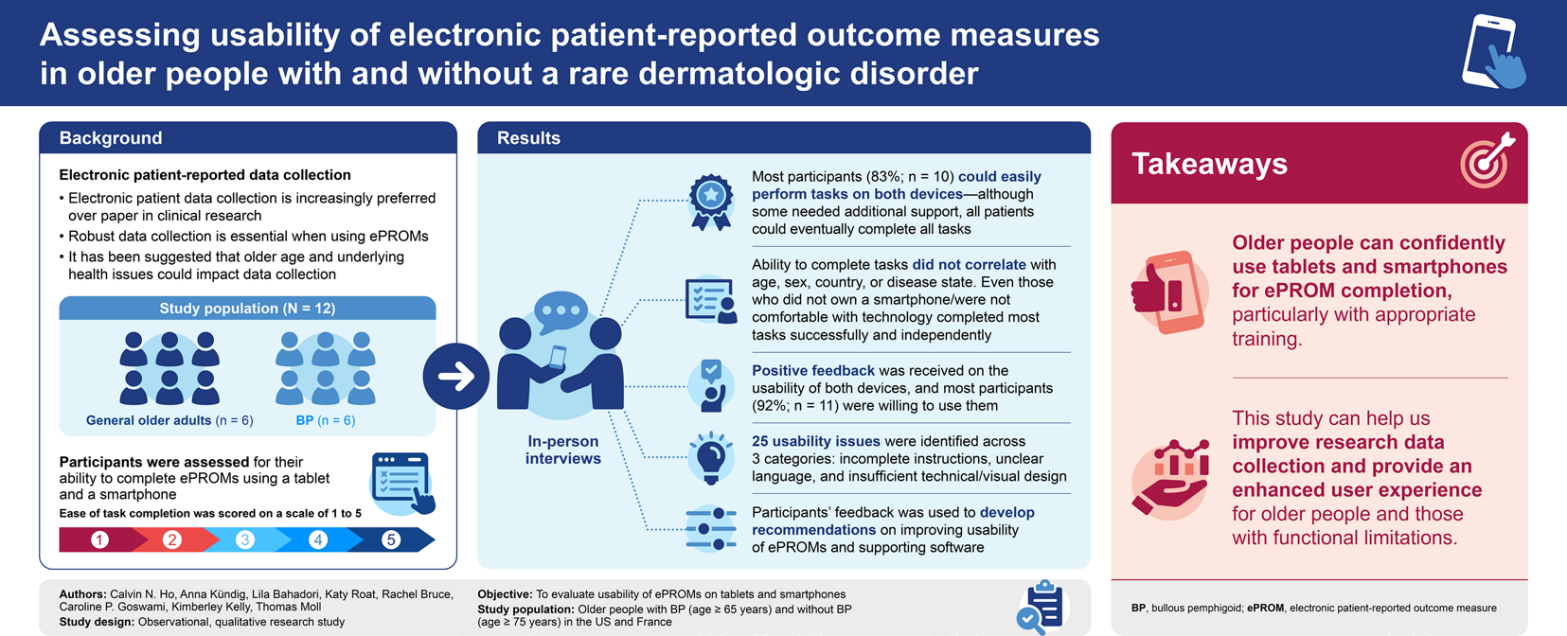

**Supplementary information:**

The online version contains supplementary material available at 10.1186/s41687-024-00821-w.

## Background

The United States Food and Drug Administration (FDA) recommends capture of clinical outcome assessment (COA) data in an electronic format [1, 2]. The advantages of electronic data collection over paper include direct entry into an electronic database, accurate time- and date-stamping, ability to set reminders, real-time data recording and transmission, remote data capture with lower frequency of in-person clinic visits, lower costs and administration time, and better data quality [2, 3].

When using electronic COAs (eCOAs) in a clinical study setting, it is vital to have a robust and well-defined setup for data collection, meet clinical trial data standards and regulatory requirements, monitor data gaps, and implement quality control and validation [4]. It is also important to identify potential barriers to uptake that should be considered when designing data collection in this format [5]. Electronic patient-reported outcome measures (ePROMs) are a type of eCOA used specifically to capture direct input from patients on their experience and perceptions of disease, health status, and functioning [6].

Older adults may have less experience with electronic devices, which could hinder effective ePROM recording in this population [3, 5]. In addition, older people are more likely to experience multimorbidity, with underlying health conditions such as neurological disorders, which may affect cognition and dexterity, impacting both paper and electronic data collection [6–8]. Certain dermatologic disorders may also interfere with touchscreen use and impact the patient’s ability to use electronic devices to record ePROMs [5, 9, 10]. Thus, older adults with certain dermatologic disorders may be hypothesized to have difficulty with ePROM completion.

Designs for ePROMs should undergo usability testing in the target population, to establish whether individuals can effectively use the device or software needed [6]. Therefore, feedback from older people is important when designing ePROMs for this population, along with sufficient training and education [6, 11]. However, limited research data are available on electronic data collection in older people in a clinical trial setting.

The present observational, qualitative research study evaluates the usability of ePROMs on tablets and smartphones in older patients with and without bullous pemphigoid (BP), a rare dermatologic disorder. The average age of BP onset is 73 years [12], and its incidence increases with age [13]. It is characterized by tense, painful blisters, which may burst under pressure, and itching across the body, including the hands [14, 15]. The aim of this study was to evaluate how well older people with or without BP could complete ePROM tasks administered on tablets and smartphones, in order to provide recommendations on how to improve usability and user experience.

## Methods

This usability interview study assessed participants’ ability to complete ePROMs using electronic devices, aiming to identify any age- or disease-related characteristics that could impact their ability to complete the tasks and to obtain participant feedback on device usability.

### Study design

The study was designed to complement a phase 3 study (FJORD; NCT04612790) in patients with BP. The FJORD study included a number of ePROMs to assess patient response to treatment, as well as an oral corticosteroid (OCS) medication usage electronic diary (e-diary) [16]. This qualitative research study consisted of structured individual one-to-one in-person interviews conducted by trained and experienced research moderators to obtain direct feedback on the ePROMs and e-diary used in the FJORD study, and to evaluate participants’ feelings on using ePROMs in a clinical study setting. An institutional review board (IRB)-approved semi-structured interview guide was used to conduct the interviews. A scoring scale ranging from 1 to 5 (1 for “unable to complete task,” 5 for “able to complete task independently, quickly, and correctly”) was used to assess success in task completion. The interview approach was in line with guidelines recommended by ISPOR Good Research Practices Task Force. The participants and interviewers had no contact prior to the interview.

### Ethical approval

The present study was conducted in accordance with the ethical principles in the Declaration of Helsinki and is consistent with Good Clinical Practice and applicable regulatory requirements. It also followed the regulations of the US FDA as described in 21 CFR 50 and 56, applicable laws, and the requirements of the WIRB-Copernicus Group IRB, as well as following the guidelines and country-specific regulations outlined in the 2022 EphMRA Code of Conduct for non-US markets.

### Recruitment

Answering the research questions in this study required in-person observational interviews. To differentiate between age- and BP-related usability issues, older adults without BP were also included.

Participants were recruited on a rolling basis in the US and France between February and June 2023 by a third-party recruiter. Two primary recruitment strategies were used: outreach to people with BP by a patient advocacy group, the International Pemphigus and Pemphigoid Foundation (IPPF), and recruitment of older participants from the general population. People interested in participating were directed to a recruiting team, who screened candidates over the phone to determine eligibility. Those who passed this screening signed an informed consent form for participation. Two screened candidates dropped out after being identified as eligible.

#### Participants

For participants with BP, the inclusion criteria were age ≥ 65 years, BP diagnosis, and no current enrollment in the FJORD trial or any other BP-related clinical trial (to ensure naivety with the ePROMs used in this study). Participants from the general population, without BP, had to be at least 75 years of age and were included to assess usability in an older age group, as recruitment of patients with BP was anticipated to be difficult due to the rarity of the disease. The average age of BP onset is 73 years [12], so the age limit for participants from the general population was set at 75 years to ensure that feedback could be captured from a representative population. Exclusion criteria for both groups were: any cognitive impairment, psychiatric condition, neurological condition, or any other clinically relevant condition that would interfere (in the screener’s opinion) with the ability to provide informed consent or participate in the study; inability to read, speak, or understand the relevant language sufficiently to complete all assessments and participate in the interview (English in the US, French in France [translated content was reviewed and quality checked]); and physical inability to complete a 60-min in-person interview or use a handheld device (including the requirement that participants be able to see the text on the screen).

The sample size was determined using established best practice for qualitative user testing. According to this, a sample of five testers is sufficient to identify most usability issues. If testing with more than one group (as in this study), three or four testers from each group are needed [17]. The population size of this study is, therefore, aligned with best practice.

### Interview methodology

Individual interviews with each participant were conducted with two moderators—one trained in moderating interviews led the discussion, while the other observed the participants’ interactions with the devices and took notes. Interviews were structured to elicit open-ended feedback and allow the interviewer to observe how participants used and physically interacted with the devices while completing tasks to evaluate usability. Participants were informed of the reasons for the study, and that it was sponsored by a pharmaceutical company.

The interviews were 60-min long and conducted in person either at the participant’s home or at a centrally located facility (see Suppl. Section 1 for additional details of how the interviews were conducted). All interviews were audio-recorded with the participants’ permission so that verbatim transcripts could be used as source data for analysis. All personal identifiable information was deleted from transcripts prior to analysis. Transcripts were reviewed and quality checked by moderators, but were not returned to participants for review.

### Data collection and study materials

The ePROM assessments tested were the Peak Pruritus Numerical Rating Scale (PPNRS), the European Quality of Life 5 Dimensions 5 Levels (EQ-5D-5L) instrument, the Bullous Pemphigoid Disease Area Index Pruritus Component (BPDAI Pruritus) items A–C, and an OCS medication usage e-diary. These assessments were used in the FJORD study as well. The tablet used was a Samsung Galaxy A7 (SM-T505) and the smartphone was a Bluebird Touch Mobile Computer (SF550); the same devices were also used in FJORD [16]. The e-diary was part of the app on the device, and included a numeric keyboard for patients to enter their OCS dosage in milligrams or an answer option to confirm that no OCS medication was taken.

The interviews followed a semi-structured interview discussion guide covering the following topics in the order listed: ePROM tablet training, task observation, and feedback; ePROM smartphone training, task observation, and feedback; and discussion on desired notifications (i.e., reminders), training materials, and other desired support (see Suppl. Section 4 for further details on study materials). This provided moderators with specific directions to give participants for each task to ensure consistency across all interviews. Moderators were instructed to not interfere or proactively provide support so as to not bias the participant’s task completion.

The ePROM tablet task observation and feedback consisted of a self-directed training module on the device, completion of two PROMs (the EQ-5D-5L and the BPDAI Pruritus tool), and an error message simulation. The training module consisted of a series of screens for the participant to click through independently that were representative of the types of response options they would encounter in the ePROMs, including a simulated error message to replicate potential user error; response option designs were a Likert scale (using radio buttons), a numerical rating scale (NRS), a visual analog scale (VAS), and a keypad for login.

The ePROM smartphone task observation and feedback began with the moderator demonstrating how to turn on the smartphone, log in (by entering the password), open the correct app, and navigate to the task menu. The participant was then asked to repeat these actions independently while the moderators observed, followed by self-led training. Next, the participant was asked to use the smartphone to complete the OCS medication usage e-diary to input a hypothetical dose of OCS into free-text fields (two scenarios: 35 mg and 0 mg, given one at a time), followed by completion of the PPNRS to rate itch for two mock scenarios: itch of 3 and itch of 8. The smartphone assessment ended with an error message simulation.

After completing each task, participants were prompted to provide feedback on how easy or difficult the task was to complete. After completing all tasks on both devices, participants were asked for more general feedback on the tablet or smartphone itself. They were asked what could potentially prevent them from completing the tasks as assigned and, for the smartphone tasks, what reminders they would need to complete the assessments daily. Finally, participants were asked how prepared they felt to complete the tasks after the training was provided, a for suggestions on the training materials and any additional desired resources.

### Data analysis

De-identified participant responses were captured via interviewer notes, an audio recording, and a written transcript. Descriptive data from the participant-reported demographics and screening documents were tabulated to characterize the sample. Participant responses were documented using an Excel-based capture grid. Each transcript was reviewed, and participant responses were analyzed for key themes related to ePROM design and usability. Themes were identified based on frequency (how often the concepts were mentioned by different participants) and severity (how significantly the given issues impacted task completion).

Moderators scored participants on their success in completing each task, using a scoring scale ranging from 1 to 5. A score of 1–2 indicated that the participant was unable to complete the task independently, while a score of 3 or higher demonstrated independent completion (1: unable to complete task; 2: required help to complete task; 3: able to complete task independently, with minor mistakes/significant hesitation; 4: able to complete task independently, with slight hesitation; 5: able to complete task independently, quickly, and correctly). Following each task, moderators asked participants how easy or difficult the task was to understand and navigate.

Moderators also noted issues that participants encountered with each task, defined as challenges that, at a minimum, caused confusion, hesitation, or delay, as expressed by the participants or observed by the moderators.

## Results

### Demographics and numbers of enrolled participants

**Fig. 1:**
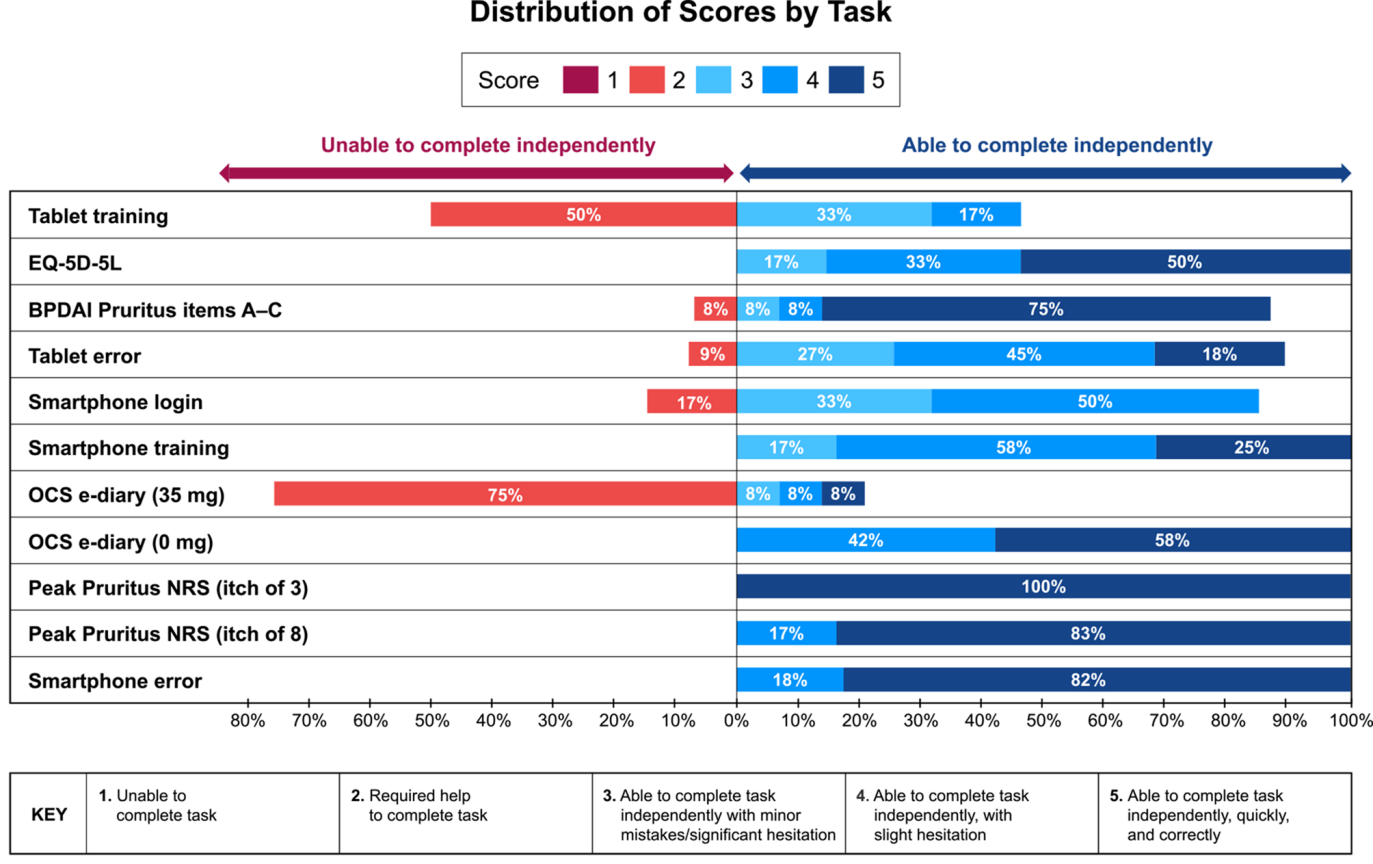
Distribution of usability scores by task. *Note* The tablet and smartphone error tasks were only tested on 11 participants, as these tasks were added after completion of the first interview

A total of 12 participants were recruited (6 each with and without BP). They came from a range of demographics, and their familiarity with technology varied considerably (Table 1). Overall, the demographics were well distributed. Participants without BP were on average 13 years older (all were >75 years old) than those with BP (65–74 years). All US participants reported owning a tablet or smartphone, while only 60% of French participants did. All participants from the general, non-BP population reported owning a tablet or smartphone, while only 67% of those with BP did (see Suppl. Section 2, Fig. 1). The difference between the US and French participants is consistent with that in published surveys; a Pew survey from 2023 found that about three-quarters of US adults aged over 65 years owned a smartphone [18], while a French government survey from 2021 found that only one-third of adults in France aged over 75 years were smartphone users [19].

**Table 1:** Demographic characteristics of usability study participants

Demographic characteristics		Participants with BP (n = 6)	General older population (n = 6)	Total (N = 12)
Age (years)^*^	65–74	6 (50%)	0 (0%)	6 (50%)
	75+	0 (0%)	6 (50%)	6 (50%)
Sex	Male	2 (17%)	3 (25%)	5 (42%)
	Female	4 (33%)	3 (25%)	7 (58%)
Country	France	2 (17%)	3 (25%)	5 (42%)
	United States	4 (33%)	3 (25%)	7 (58%)
Tablet/smartphone ownership	Yes	4 (33%)	6 (50%)	10 (83%)
	No	2 (17%)	0 (0%)	2 (17%)
Comfort with technology	Not at all	1 (8%)	0 (0%)	1 (8%)
	Somewhat	3 (25%)	4 (33%)	7 (58%)
	Very	2 (17%)	2 (17%)	4 (33%)

*BP* bullous pemphigoid

^*^Age cut-offs were established as part of the eligibility criteria

### Summary of device usability

All participants successfully completed all tasks on both the tablet and smartphone. Most participants were also able to complete the tasks independently (n = 10; 83% [usability score ≥ 3]), and issues were triaged for impact on task completion, as well as for frequency (see Suppl. Section 3). Exceptions were the tablet training and the first part of the OCS medication usage e-diary, where 83% (n = 10) and 75% of participants (n = 9), respectively, needed assistance. The breakdown of participant scores by task is shown in Fig. 1. A score of 3+ (shown in blue) indicates independent task completion. There were no major differences in usability score by participant age, sex, country, disease state (BP or no BP), or previous experience/comfort with tablets or smartphones, suggesting that lack of experience or comfort with a tablet and/or smartphone does not translate to an inability to complete ePROMs. This is supported by the ease with which participants who did not own a smartphone (n = 2) were able to complete most tasks.

### Issues encountered with ePROM device use

#### Tablet issues

Issues encountered with each task are summarized in Table 2, with additional data outputs provided in Suppl. Section 4. During the tablet training task, half of the participants (n = 6; 50%) needed assistance at some point. The main issues preventing task completion were related to a lack of clear on-screen instructions and ambiguous questions. For example, commenting on a sample question with non-substantive answer options, one participant said: “Doesn’t make sense to me right now … I don’t know what the 6 options are … I’m supposed to select an option, but these 6 options don’t have any value.” The EQ-5D-5L task was generally viewed by the participants as easy to complete, both in terms of understanding the task and physical navigation, although one participant indicated that they had expected a sliding scale for the VAS implementation. No issues were identified that prevented participants from completing the task independently. The BPDAI Pruritus task was considered easy to complete by 83% of participants (n = 10), both in terms of understanding and physical navigation. However, upon advancing from the first to the second screen, three participants did not realize that they had proceeded to a new screen and required assistance to proceed. One of these participants said: “I didn’t realize it had changed. If you hadn’t seen that the question had changed, I’d have been stuck pressing it like a fool.”

**Table 2:** Summary of issues occurring with the tablet (issues are listed in the order in which they were encountered)

Task	Issue description	Frequency of occurrence	Did issue prevent any participants from completing the task independently?	Common to both tablet and smartphone?
Tablet training	Participants do not know to click the drop-down bar	42% (n = 5)	Yes	No
	Participants do not know which option to select from the drop-down menu	67% (n = 8)	Yes	No
	Participants are confused by ambiguous mock questions	50% (n = 6)	Yes	Yes
	Participants are confused by the task requiring them to go “back”	42% (n = 5)	No	No
	Two different words are used for “back” on the French tablets	17% (n = 2 of 5 French participants)	No	No
	Participants are unsure how to close the training module	42% (n = 5)	Yes	Yes
	Participants are inclined to close the training video prematurely	8% (n = 1)	No	No
EQ-5D-5L	Participants struggle to use the VAS scale of the EQ-5D-5L	33% (n = 4)	No	No
	Participants do not realize the question has changed	8% (n = 1)	No	No
BPDAI Pruritus Component items A–C	Participants do not realize the question has changed	25% (n = 3)	Yes	No
Error message*	Error message is unclear	64% (n = 7)	Yes	Yes
	Error message disappears too quickly	45% (n = 5)	No	Yes

See Suppl. Section 4.1 for screenshots of issues encountered with tablet use

*Only 11 of the 12 participants were tested on the error task, as the error message task was not added to the discussion guide until after the first participant had been interviewed

The tablet error message task was not added to the discussion guide until after the first participant had been interviewed. Therefore, only 11 participants were assessed, and it was independently completed by 10 (91%) of them. Seven (64%) participants said the tablet error message was unclear, five said it disappeared too quickly (45%), and seven (64%) initially clicked “back” on seeing the tablet error message rather than selecting an answer to proceed (see Suppl. Section 4.1, Figure 10). Participants also noted that it would be helpful if the error message specified what was wrong and what they needed to do differently.

#### Smartphone issues

Issues with smartphone use are summarized in Table 3. During the login task, 10 participants (83%) were able to log in without assistance. One required direction on where to enter the password. Four (33%) participants had difficulty swiping to unlock the smartphone, and one eventually required the moderator’s assistance. All participants were able to complete the smartphone training independently. Participants experienced difficulty with the first scenario in the OCS medication usage e-diary, with nine (75%) requiring assistance to complete the task. Four got temporarily stuck because they wanted to label their entry “mg” and could not find a letter keyboard. One said: “I wanted to put ‘milligrams,’ but the letters have to appear, I only have numbers. I don’t know [how] to get to the letters.” Eight (67%) were unable to proceed due to the “Next” button being hidden behind the numeric keyboard on the screen, with one commenting: “I got stuck because ‘Next’ wasn’t available.” However, once they got past these issues, all participants were able to complete the second scenario without assistance and generally described this task as easy to complete, possibly because they could easily understand what they were required to do.

**Table 3:** Summary of issues occurring with the smartphone (issues are listed in the order in which they were encountered)

Task	Issue description	Frequency of occurrence	Did issue prevent any participants from completing the task independently?	Common to both tablet and smartphone?
Smartphone login	Participants struggle to swipe to unlock the smartphone	33% (n = 4)	Yes	No
	Participants do not know where to enter the password	8% (n = 1)	Yes	No
	Participants struggle to find the “log in” button	25% (n = 3)	No	No
Smartphone training	Participants struggle to navigate the keyboard	42% (n = 5)	No	No
	Participants are confused by ambiguous mock questions	8% (n = 1)	No	Yes
	Participants do not know how to close the training module	8% (n = 1)	No	Yes
OCS medication usage e-diary	“Next” button is hidden by numeric keyboard	67% (n = 8)	Yes	No
	Participants try to input unit of measurement	33% (n = 4)	Yes	No
	Participants click the checkbox unnecessarily	33% (n = 4)	Yes	No
	Participants do not mark the checkbox when indicating they did not take OCS*	25% (n = 3)	Yes*	No
	Participants struggle to open the numeric keyboard	8% (n = 1)	No	No
Smartphone error message^†^	Error message disappears too quickly	36% (n = 4)	No	Yes
	Error message phrasing is unclear	9% (n = 1)	No	Yes

See Suppl. Section 4.2 for screenshots of issues encountered with smartphone use

*This issue was defined as preventing independent progress, even though participants were able to proceed independently, because this issue could result in inaccurate data depending on how a “0” in the OCS e-diary is handled in data processing

^†^Only 11 of the 12 participants were tested on the error task, as the error message task was not added to the discussion guide until after the first participant had been interviewed

All participants (N = 12) achieved a perfect usability score in the PPNRS task, describing it as “very simple” to complete. Eleven participants took part in the smartphone error message task (see Suppl. Section 4.2, Figure 8) and completed it independently, despite the issue of the error message disappearing too quickly for some participants to read and comprehend (n = 4; 36%). One participant was confused by the phrasing of the error message (which stated, “Please respond”), suggesting: “There has to be a message here that says, ‘We need your response,’ instead of ‘Please respond,’ because you think you did respond.”

### General device feedback

#### General feedback on the tablet

Overall feedback on the tablet was positive; three (25%) participants liked that it was small and lightweight, making it easy to hold and maneuver. Two (17%) appreciated that the screen was bright, which made the content easy to see. Four participants (33%) suggested that the font size should be increased (specifically for the response options on the EQ-5D-5L and the scoring components on the BPDAI Pruritus tool). The text was deemed readable by all participants, but some needed to put glasses on to complete the tasks.

#### General feedback on the smartphone

Overall feedback on ease of understanding and physical navigation was positive. Five participants (42%) mentioned that prior familiarity with smartphones gave them the confidence and transferrable skills to successfully use the study device. Four participants (33%) suggested that the font size should be increased, although all participants could read the text, indicating that a small font size is unlikely to prevent task completion.

### Participant feedback and suggestions on ePROM device use

Almost all participants (n = 11; 92%) expressed willingness to use electronic devices for completing ePROMs in a clinical study setting, with only one expressing a preference for pen-and-paper questionnaires. Participants did not have a strong preference for either tablet or smartphone, but when prompted, six participants (50%) expressed a preference for the tablet, citing the larger screen as easier to see and read. One participant additionally mentioned the tablet being harder to lose as an advantage (n = 1; 8%). Four participants (33%) expressed a preference for the smartphone, stating that it was easier to carry and hold as it was smaller, while two participants (17%) stated no preference.

#### Potential barriers to tablet tasks

Seven participants (58%) indicated that nothing would prevent them from completing the same tasks in the context of a site visit. Two participants (17%) noted that remembering how to complete the tasks could be a barrier to participation, and one mentioned (8%) that sickness/depression could be a barrier.

#### Potential barriers to smartphone tasks

Only one participant (8%) stated that they would not complete tasks every day, while nine (75%) were certain that they would. Forgetting (n = 4; 33%), experiencing a severe medical event (n = 1; 8%), and poor internet connection (n = 1; 8%) were cited as potential factors interfering with task completion. One participant with BP noted that even having a BP flare would not prevent task completion, stating: “If I was sick and I needed this, I would do it.”

See Suppl. Section 5 for potential barriers in a clinical study setting.

#### Reminders

Participants were asked what would help them remember to complete smartphone tasks (tablets usually remain at study sites, so are not applicable here). Suggestions included establishing a routine (n = 8; 67%), receiving an audible alarm (n = 8; 67%), receiving an electronic notification at a fixed time each day (n = 7; 58%), receiving electronic reminders to personal devices (n = 7; 58%), keeping the phone in a visible place (n = 2; 17%), receiving follow-up reminders until the task is completed (n = 1; 8%), and receiving reminders that require a response by mid-afternoon (n = 1; 8%).

#### Training and support

Overall, participants were pleased with the training modules offered on both devices, describing the training as sufficient, straightforward, and helpful. One participant (8%) emphasized the value of having an in-person trainer to demonstrate the tasks and provide support. Participants’ recommendations included being offered the opportunity to repeat the training at any point in the study journey, a 24-h live helpline (n = 6; 50%), a printed instruction booklet or summary (n = 5; 42%), an online chat/website (n = 1; 8%), and additional “smartphone 101” training for those who do not have experience using a tablet or smartphone (n = 1; 8%).

### Recommended solutions to identified issues

All issues (both those preventing independent task completion and those causing confusion or hesitation but not preventing completion) were assessed, and relevant recommendations were provided based on participants’ feedback and interviewers’ observations. These can be used to improve digital engagement in future studies; see Suppl. Section 6, Table 6.1 for more details.

## Discussion

The aim of this study was to understand how well participants could complete ePROM tasks administered on tablets and smartphones, and to provide recommendations on how to improve usability and user experience. We found that most participants were able to easily and confidently use a tablet or smartphone for most assigned tasks.

All participants in this study were able to complete all assigned tasks, although some required assistance to do so. Ability to complete the assigned tasks did not correlate with participants’ age, sex, country, or disease state (BP or no BP). All identified issues were due to fixable software design elements or ambiguous wording in the on-screen instructions. Certain elements posed challenges on the tablet but not on the smartphone, suggesting that participants were able to transfer their learnings from the tablet tasks. This indicates that, while some people may require guidance in initially familiarizing themselves with the devices, it is feasible even for those who are less tech-savvy to complete ePROMs autonomously within the framework of clinical trials. When specifically asked, participants were generally receptive to using tablets and smartphones to complete ePROMs in clinical trials, giving positive feedback on both, and most were willing to use them, as completion was seen as part of their healthcare. Further, participants expressed confidence in their ability to consistently complete the tasks, although they did mention that establishing a routine and receiving reminders would be helpful in maintaining compliance.

Of the 25 usability issues identified during this study, 12 were deemed severe enough to potentially prevent task completion, mainly due to unclear instructions, confusing language, or unintuitive software design. Recommendations on how to overcome specific issues are detailed in the supplementary material (Suppl. Section 6, Table 6.1). The feedback provided by study participants has led to the development of general principles regarding usability, training, and compliance. These insights, summarized in Suppl. Section 6, Table 6.2, may have the potential to enhance the future design of software supporting eCOA completion (including ePROMs) for older users. Notably, providing substantive content in place of mock questions was a key finding that impacted most participants interviewed and has implications for how software used to present eCOA training materials should be developed. The feedback received on text size and readability is particularly relevant when considering participants with BP, as this population may be taking corticosteroids, which can be associated with ocular complications [20].

The BP patient population largely consists of older people, but more than 90% of the sample population in this study had no reservations about the use of electronic devices (Suppl. Section 7) and found them straightforward to use (Suppl. Sections 7 and 8). Older patients may also experience age-related difficulties such as poor eyesight and hearing, memory loss [11], and underlying medical conditions [7, 11] that could influence the execution of data collection tasks using paper or electronic formats [5, 6]. The use of technical features such as scale implementation and zooming to support accessibility and usability is recommended when developing and implementing the software used to present ePROMs [21]. Recently released recommendations for PRO use in clinical practice highlight the potential burden that PRO completion can place on respondents and outline ways of reducing this, namely, ensuring that respondents and clinicians be involved in decisions, that PROs used are relevant for the target population, and also that respondents and clinicians are involved when updating or developing new PRO measures [22]. Another consideration is that many PROMs were originally developed and validated in a paper format, and migration to an electronic format needs to be undertaken with careful consideration to preserve the integrity of data collection. Best practices have been established to ensure equivalence between the paper and electronic formats and should be considered when developing ePROMs and the supporting software for device use, but there is a continuous need to identify and action the most optimal solutions for successful implementation [23]. This is supported by a recent study [24] in patients with cancer aged ≥75 years, which showed a limited feasibility rate (26%) associated with remote monitoring of ePROMs, mainly due to technological barriers. A higher rate of use (66%) was seen in patients who had Internet access, compared with the overall study population, and the authors concluded that overcoming technological barriers was important to improve care in these patients [24]. Although eCOAs (including ePROMs) should follow industry best practices to maintain scientific validity, usability guidelines for the supporting software have not been developed. In the absence of formal guidelines, it is important to ensure that such software is adapted to support effective use of the eCOA/ePROM by the target patient population [6, 21, 23].

The results of the present study show that older people can effectively use a tablet and smartphone for ePROM completion with proper training, which is consistent with previous research that showed positive attitudes toward mobile technology among older people (mean age, 73 years) [25]. The present study highlights the importance of providing clear instructions, using simple mechanics, and offering training. This aligns with the previous research, which had demonstrated that interviewed participants preferred simplicity when completing ePROMs and emphasized the significance of providing adequate training [25].

There remains a need to work with people to meet their needs and reduce barriers to technology use, so that study and patient-reported data can be seamlessly recorded and integrated across different platforms within the context of clinical trials [24, 26]. A considerable obstacle to the adoption of technology is the lack of input from older people and patients in the design of eCOA technologies and services, which may result in failure to address the right issues and concerns for these populations [26]. The findings of the present study add to our understanding of the technology needs of older people and of how to enhance eCOA software and device usability to improve patient-reported data capture.

### Study limitations and general applicability

Although the interview questions are relevant to all devices and patient populations, the small sample size may impact the generalizability of the study findings to other patient populations and other types of eCOA devices designed to capture ePROMs. The questions and concerns explored are potentially applicable to all devices and for a broader patient population.

Due to these limitations, further research is warranted to test ePROM usability in a larger population of older people and to assess how prior technological familiarity may affect user experience with ePROMs. Additional research is needed in rare diseases, as well as in other sub-populations that may have symptoms that could limit the usability of eCOAs, including ePROMs. It is important to proactively identify such patients and consider their specific limitations in the design process for eCOAs and ePROMs to help mitigate usability issues.

## Conclusions

In the present study, older participants were able to confidently complete ePROM tasks on both a tablet and a smartphone. Identified usability issues were largely due to amendable design elements and were unrelated to participant age, sex, country, or disease state (BP or no BP). The overall feedback on using these devices to complete a series of tasks was positive, with most participants expressing their willingness to use them.

eCOA devices have revolutionized the way clinical trials are conducted, providing a more accurate and efficient means of collecting patient-reported outcomes. However, concerns have been raised about the usability and accessibility of these devices for older people, who may have limited technological skills and/or functional impairments. Insights gained from this study can shape future best practices for the development of user-friendly ePROMs that will ultimately aid in informed decision-making and improve patient outcomes.

## Electronic supplementary material

Below is the link to the electronic supplementary material.

Supplementary Material 1

## Data Availability

Data underlying the findings described in this manuscript may be obtained in accordance with AstraZeneca’s data sharing policy described at: https://astrazenecagrouptrials.pharmacm.com/ST/Submission/Disclosure.

## Abbreviations

BP: Bullous pemphigoid
BPDAI: Bullous Pemphigoid Disease Area Index
COA: Clinical outcome assessment
eCOA: Electronic clinical outcome assessment
ePROM: Electronic patient-reported outcome measure
EQ-5D-5L: European Quality of Life 5 Dimensions 5 Levels instrument
FDA: Food and Drug Administration
NRS: Numerical rating scale
OCS: Oral corticosteroid
PPNRS: Peak Pruritus Numerical Rating Scale
PROM: Patient-reported outcome measure
US: United States
VAS: Visual analog scale
