# Supplementary material for: Assessing usability of electronic patient-reported outcome measures in older people with and without a rare dermatologic disorder
Source: J Patient Rep Outcomes. 2024 Dec 18;8:145. doi: 10.1186/s41687-024-00821-w (PMC11655768; doi:10.1186/s41687-024-00821-w)
Supplement: Supplementary file 1 — Supplementary Material 1 [file 41687_2024_821_MOESM1_ESM.docx]

## **SUPPLEMENTARY MATERIAL**

### **Section 1: Patient interviews—additional methodology details**

Interviews were structured to elicit open-ended feedback and allow the interviewer to observe how participants used and physically interacted with the devices while completing tasks to evaluate usability. The interviews were 60-minutes long and conducted in person either at the participant’s home or at a centrally located facility (ie, a hotel conference room) at various locations throughout the US and France. The locations were selected based on patient choice, and all except one chose to have the interview conducted at their home. The devices used, tasks completed, and methodology used were the same at all locations. All interviews were audio-recorded with the participants’ permission so that verbatim transcripts could be used as source data for analysis. All personal identifiable information was scrubbed from the transcripts prior to analysis.

### **Section 2: Additional study data demographics**

#### 2.1 Participant familiarity with technology

*Figure 1: Percentage of participants who reported owning a personal tablet or smartphone*


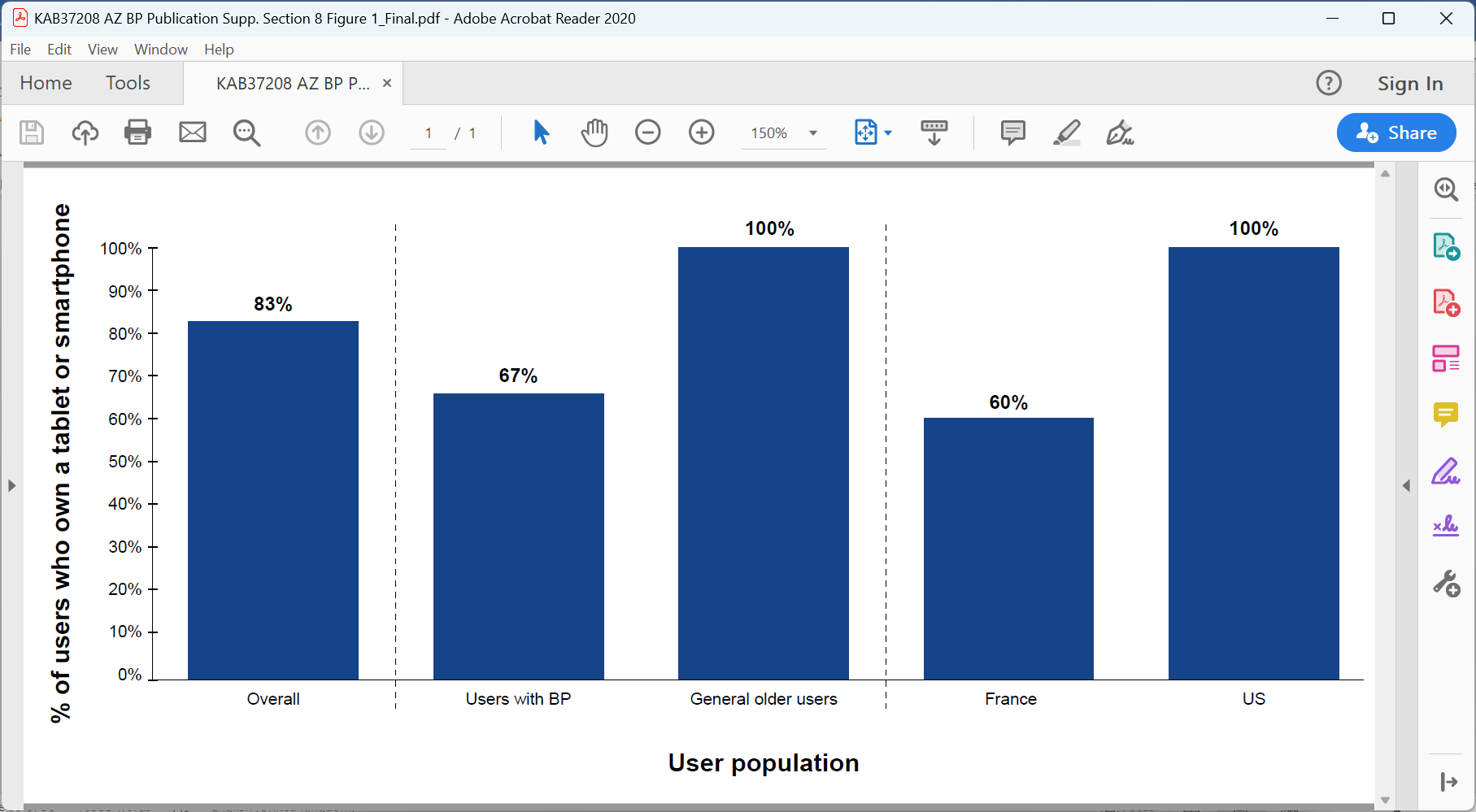


*Figure 2: Self-reported comfort with technology*


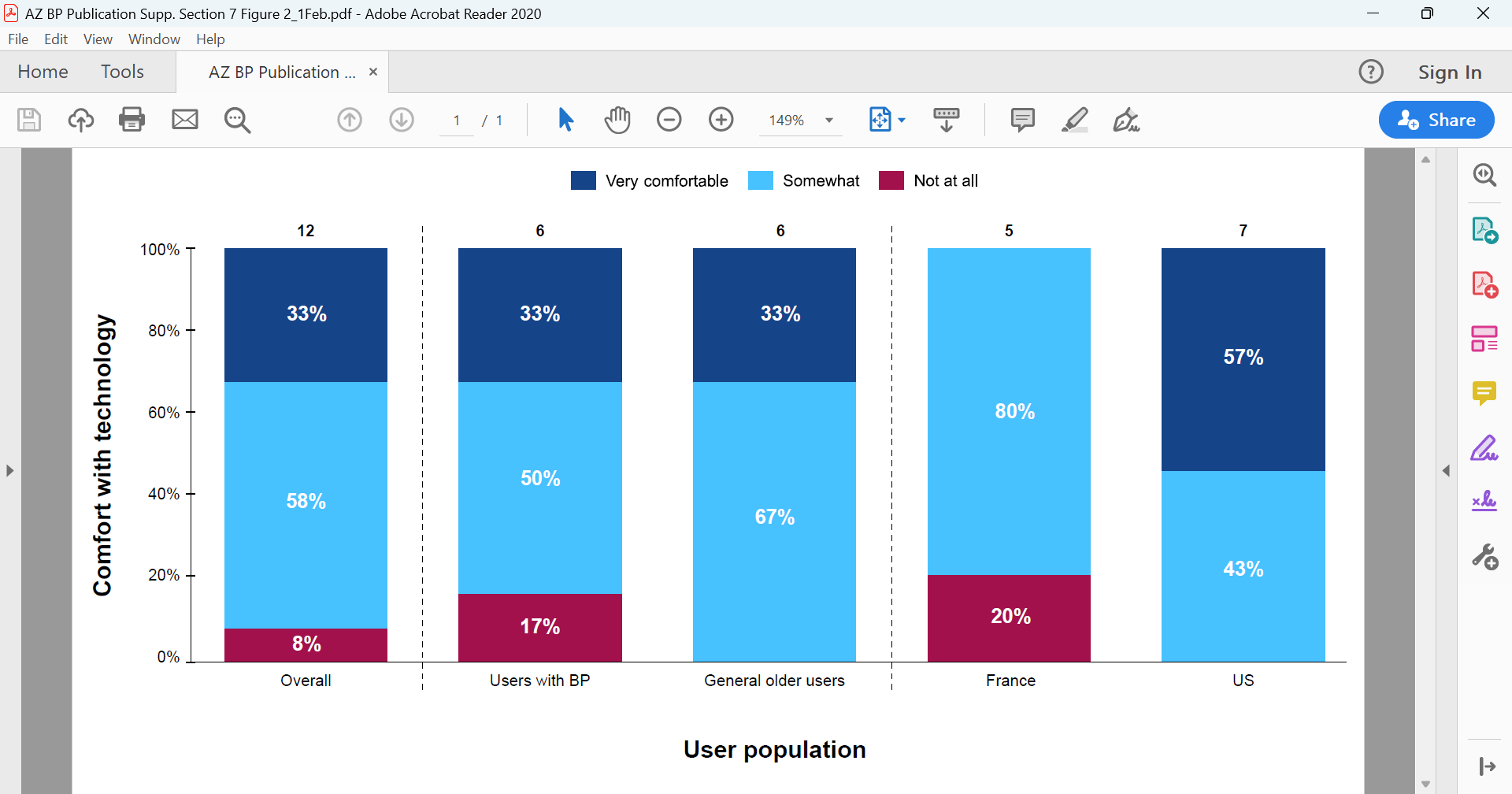


### **Section 3: Issue triage matrix figure**


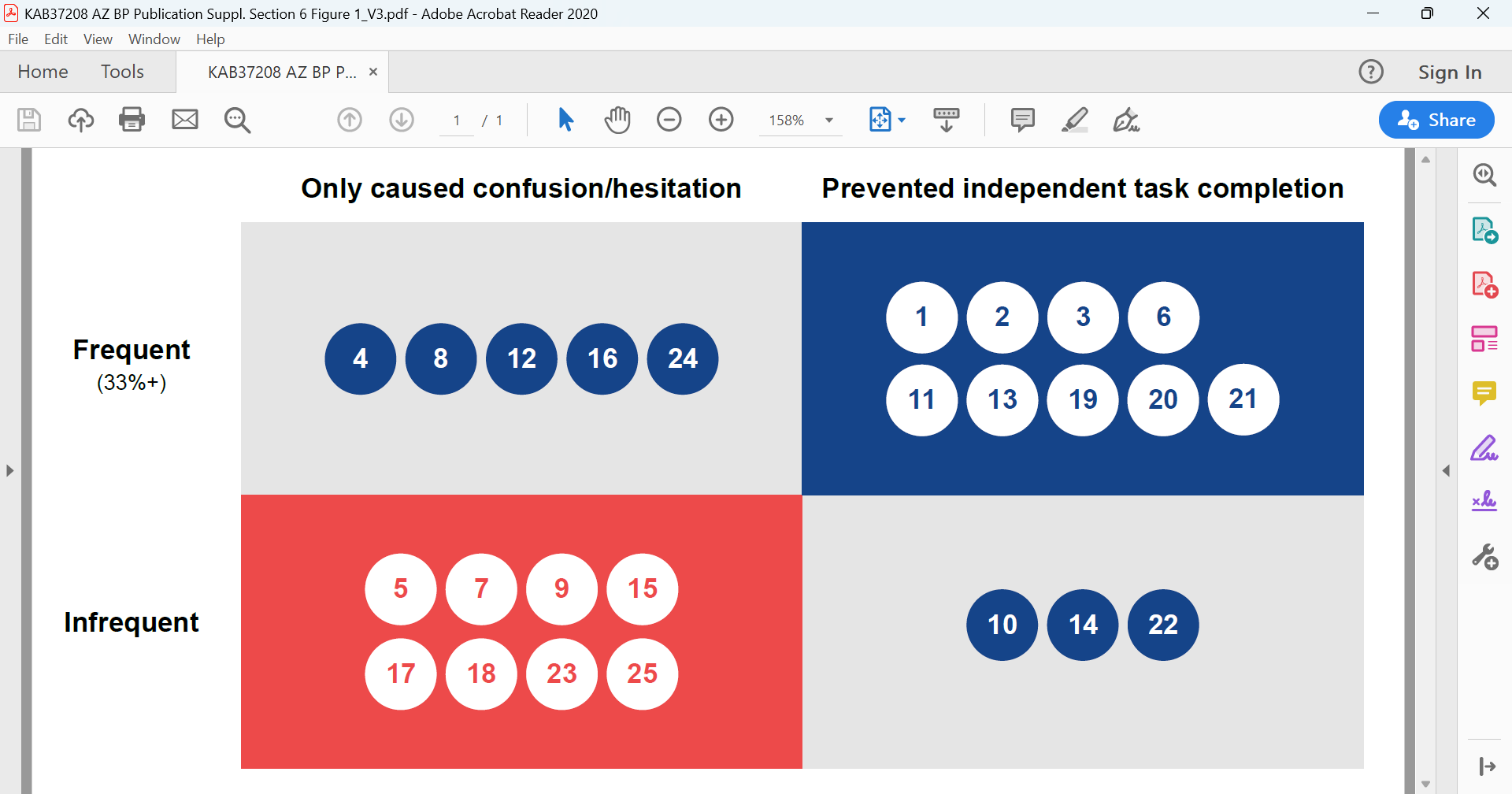


### **Section 4: Additional study data outputs—details of identified issues and functionality**

Additional details and screenshots are provided here to clarify the nature of the issues encountered by participants, and how they appeared on the screens.

#### 4.1 Issues encountered: Tablet

Most issues in the tablet training occurred due to a lack of clarity regarding the questions being asked.

*Figure 1: Participants do not know to click the dropdown bar*

The tablet training module screen prompted participants to select an option from the dropdown menu:

Nearly half of the participants (n = 5; 42%) did not know to click the bar to open this. Participants were confused because the instructions said to “tap on the box below,” and they did not see what they perceived to be a “box.”

*Figure 2: Participants do not know which option to select from the drop-down menu*

On the dropdown menu screen, participants (n = 8; 67%) were initially confused as they did not know to select an option (n = 6; 50%) or did not know which option to select (n = 4; 33%). Two (17%) participants were confused by both. Participants were intent on providing accurate responses and were confused by the ambiguity of the prompt (no question provided) and response options that lacked value.

*Figure 3: Participants are confused by ambiguous mock questions*


Half of the participants (n = 6; 50%) were confused by the non-specificity of two questions in the tablet training module: “How satisfied are you?” and “How often do you feel pain?”. Participants did not understand that these questions were meant to be mock questions and asked the moderator for clarity before knowing how to proceed.

*Figure 4: Participants are confused by the task requiring them to go “back”*

Several participants (n = 5; 42%) were confused by the portion of the tablet training module that asked them to tap the “back” button a few times and then tap the “next” button to return to the current screen. They did not know how far back to go or were confused as to why they were seeing the same screens again. However, all participants were eventually able to proceed independently.

*Figure 5: Two different words are used for “back” on the French tablets*

On the French version of the tablet training module, the screen that asked participants to tap the “back” button a few times used a different word for “back” in the instructions (“retour”) than what was written on the button on the screen (“précédent”). While this difference did not prevent any participants from completing the task, it did cause two (40%) participants to pause in confusion.

*Figure 6: Participants are unsure of how to close out of the training module*

On the last screen of the tablet training module, participants were presented with two options:

they were asked whether they would like to “finish the training module” or start it again. Multiple participants (n = 5; 42%) did not know which they should select to complete the training and move on to the next task. One participant (8%) did not realize they were already at the end of the training module, so they did not know whether to “finish” or “start,” and they required moderator direction. Another (n = 1; 8%) thought they were being asked to complete the module again.

*Figure 7: Participants are inclined to close the training module prematurely*

Participants were inclined to click the “close” button as soon as it popped up on the final screen of the tablet training video, about 15 seconds before the voiceover audio finished. Nearly every participant began to move their finger to click the “close” button upon first seeing it, although only one (8%) did close the training prematurely. This is not a critical issue, as these 15 remaining seconds do not contain essential instructions, and this did not prevent any participants from proceeding.

*Figure 8: Participants struggle to use the EQ-5D-5L response scale*

On the final page of the tablet EQ-5D-5L, participants were asked to tap on a scale to rate their health for that day. One-third of the participants (n = 4; 33%) had difficulty initially selecting their desired number, with two (17%) trying to slide their finger rather than tapping. All were eventually able to select the desired number and proceed without assistance. One (8%) noted that they expected a slider since that is what they have encountered on questionnaires like this before.

*Figure 9: Participants do not realize the question has changed (EQ-5D-5L; a. first screen, b. second screen)*

**

After advancing from the first to the second screen in the tablet EQ-5D-5L, one participant (8%) initially did not realize that they had moved on to a different question. They thought they had been brought back to the same question they had just completed, so they attempted to click “next” without selecting an answer. After receiving an error message, they realized independently that they needed to select an answer to proceed.

*Figure 10: Participants do not realize the question has changed (BPDAI pruritus tool; a. first screen, b. second screen)*

**

After advancing from the first to the second screen of the BPDAI Pruritus tool on the tablet, three participants (25%) did not realize that they had proceeded to a new screen. Two (17%) initially tried to proceed to the next screen without selecting a response. One (8%) stared at the screen, not realizing the question had changed, until the moderator told them that it was a new question.

*Figure 11: Tablet error message*

To simulate the tablet’s error message, moderators instructed participants to pretend that they had intended to select a given number but did not do so successfully and to attempt to proceed to the next screen. This generated the error message shown below. Participants were asked how they would interpret the message, and what they would do if they encountered such a message.

All participants but one (91%) were able to complete this task without assistance. While multiple participants said the tablet error message was unclear (n = 7; 64%) or disappeared too quickly (n = 5; 45%), only one (9%) participant could not complete the task independently because the error message did not provide clear enough instructions. This error message task was only tested on 11 of the 12 participants, as it was not added to the interview guide until after the first interview.

Most participants (n = 7; 64%) initially clicked “back” upon seeing the tablet error message, rather than selecting an answer and proceeding. They interpreted that something was done incorrectly due to the red outline, but their instinct was to click “back” rather than to select an answer. Upon receiving the “are you sure you want to exit?” message, most (n = 6; 55%) knew to return to the screen and select an answer. One (9%) needed to be explicitly directed to select an answer.

#### 4.2 Issues encountered: Smartphone

*Figure 1: Participants do not know where to enter the password*

After unlocking the smartphone, one participant (8%) did not know where to enter the password and required assistance from the moderator.

*Figure 2: Participants struggle to find the “log in” button*

After entering the password, participants needed to click the “Log in” button above the keyboard. Three (25%) participants had difficulty finding this button and were unsure of how to proceed; all three were able to eventually find and click the button without help.

*Figure 3: Participants struggle to navigate the smartphone keyboard*

At one point in the handheld training module, participants were asked to enter a number in a box below using a numeric keyboard. At first, the screen appears with a blank bar and no keyboard. When the participant taps on the bar, a numeric keyboard opens. Three participants (25%) did not initially know how to proceed upon encountering the first screen because they did not know to click on the data entry bar to open the keyboard. All eventually figured out how to proceed independently.

*Figure 4: Participants are confused by ambiguous mock questions*

One participant (8%) was confused how to respond to the mock question “How satisfied are you?” because it was unclear what specifically the question was referring to. The participant did not understand that it was a mock question.

*Figure 5: Participants do not know how to close the training module*

At the end of the handheld training module, one

participant (8%) selected “Start this training module again” instead of “Finish the training module,” which took them back to the beginning of the training. The participant did not realize they had already completed the module and thought they were just beginning it.

*Figure 6: “Next” button is hidden by the numeric keyboard*

The most prominent difficulty faced by participants when completing the OCS medication usage e-diary was that after entering the OCS dosage, the “Next” button was not visible because it was covered by the numeric keyboard. Eight participants (67%) were unable to proceed because they could not figure out how to find the “Next” button. They all emphasized the importance of having the “Next” button visible at all times, even when the numeric keyboard is open.

*Figure 7: Participants click the check box unnecessarily*

The screen showed a checkbox that participants were only meant to select if they had not taken any steroid in the past 24 hours. However, when prompted to indicate they had taken 35 mg in the past 24 hours, four participants (33%) automatically selected the checkbox without reading the text carefully. This then removed any text that the participants had previously typed in the data entry field, causing further confusion, as they did not understand why the number they had just entered disappeared. One participant (8%) had to be explicitly directed to re-enter the “35” and click “next” instead of checking the blue check box; the others (n = 3; 25%) figured this out independently. This issue could lead to inaccurate data.

Participants’ default behavior is to check a box when available. This may be exacerbated by the fact that participants were asked to check a box at the end of each task to verify their information is correct and that they would like to proceed, so participants are conditioned to automatically select blue checkboxes like the one shown here.

*Figure 8: Smartphone error message*

Moderators simulated a smartphone error message by asking participants to pretend that they intended to select an answer on the Peak Pruritus NRS but were not successful in selecting a response, and then to click “next” to proceed to the next screen. Upon doing this, an error message appeared as a red bar along the top of the screen with the message “Please respond.” Participants were asked how they would interpret this message and how they would respond.

All participants (n = 11) were able to complete this task independently, although two minor issues were encountered:

- Error message disappears too quickly
  - The primary issue with the smartphone error message is that it disappears too quickly for participants to read and comprehend it. Four participants (36%) faced this issue.
- Error message phrasing is unclear
  - One participant (9%) from the US was confused by the phrasing of the smartphone error message and suggested, “We need your response” as alternate phrasing. The use of the phrase “please respond” made this participant think that they were being asked to repeat the module again, incorrectly thinking they had already responded to the question on the screen. However, they were able to proceed independently.

This error message task was only tested on 11 of the 12 participants, as it was not added to the interview guide until after the first interview.

#### 4.3 Tablet general feedback

The device was described as “easy,” “comfortable,” and “simple” to use. One participant (8%) who expressed a general aversion to using devices in day-to-day life noted that the tablet was easy to use once they understood how to use it. In terms of constructive feedback, a few (n = 4; 33%) participants suggested that the font size could be larger (specifically, the answers on the EQ-5D-5L and the BPDAI Pruritus items). All participants were able to read the text, but several had to put on glasses, and others commented that the font could be too small for other users to read. This feedback is particularly relevant for the patient with BP, as they may take corticosteroids such as prednisone, which can cause vision loss. Several participants (n = 8; 67%) also had to tap the “next” button on various screens multiple times to get a response. However, this did not cause frustration and was primarily observed during the training module, indicating that participants simply needed to familiarize themselves with the device.

#### 4.4 Smartphone general feedback

Overall feedback was positive, both from an understanding and physical navigation perspective. In particular, participants were pleased with the small size and light weight of the phone, with several (n = 5; 42%) participants commenting on how it was easy to hold and physically maneuver. Giving constructive feedback on the smartphone device, four (33%) participants suggested an increase in font size. Specifically, one participant suggested increasing the font of the text under the OCS e-diary checkbox (“Over the past 24 hours, I did not take a dose of oral corticosteroid medication”). However, all participants were able to successfully read the text.

### **Section 5: Recruitment drivers and barriers**

Only participants with BP (n = 6) were interviewed for their potential interest in a hypothetical trial similar to FJORD. Of these, four expressed potential interest, mentioning certain conditions for participation, including active disease, close proximity to the trial site (within their metropolitan area), steroids not being mandatory, receiving sufficient information about the study and study medicine, and ability to withdraw from the trial as desired. Relief from symptoms and the desire to help others were noted as reasons for interest, while fear of side effects or uncertainty around study drug reactions and concerns about travel distance were deterrents.

Ten participants from the full set were asked whether the required use of ePROMs would deter them from participating in a clinical trial (responses were not collected for two French participants from the general population, as this question was included after they were interviewed). Eight confirmed that the requirement to use a tablet and smartphone would not be a barrier, one indicated that they would not participate under this requirement, and one clarified that additional support might be needed.

### **Section 6: Final outputs and recommendations for improvement**

#### Supplementary Table 6.1: Issue description and recommended solutions

| **Task** | **Issue description** | **% participants impacted by issue** | **Did the issue prevent any participants from completing the task independently?** | **Recommended solution(s)** |
| --- | --- | --- | --- | --- |
| Tablet training | 1. Participants do not know to click the dropdown bar | 42%  (n = 5) | Yes | Adjust the wording to say, “tap on the bar below” or “tap on the arrow below” rather than “tap on the box below”, as users do not  perceive the drop-down bar as a “box.” |
|  | 2. Participants do not know which option to select  from the dropdown menu | 67%  (n = 8) | Yes | Add a substantive, non-ambiguous question with meaningful response options in place of “option 1, option 2, etc.” |
|  | 3. Participants are confused by ambiguous mock  questions | 50%  (n = 6) | Yes | Add specificity to the question, such as “How satisfied are you **with the weather** today?” or “How happy are you right now?” |
|  | 4. Participants are confused by the task requiring them to go “back” | 42%  (n = 5) | No | Provide specific instructions like: “Tap the ‘back’ button once to return to the previous screen. Then tap ‘next’ to return to this screen. Then tap ‘next’ to proceed to the following screen.”  Alternatively, delete this screen altogether. Even tech-naive participants understood the “next” and “back” buttons. |
|  | 5. Two different words are used for “back” on the French tablets | 17%  (2 of 5 French participants) | No | Ensure the text in the instructions match the text on the buttons, ie, replace “retour” with “précédent” in the instructions. |
|  | 6. Participants are unsure of how to close out of the  training module | 42%  (n = 5) | Yes | Replace existing question and answers with: “You have completed the training module.  Would you like to A) Continue or B) Re-do the training module” |
|  | 7. Participants are inclined to close the training video  prematurely | 8%  (n = 1) | No | Do not allow the “Close” button to appear until the voiceover has finished. |
| EQ-5D-5L | 9. Participants struggle to use the EQ-5D-5L scale | 33%  (n = 4) | No | Make the scale larger (if possible) and/or offer participants a stylus.  Bold the word “tap” in the instructions, so users do not attempt to slide along the scale. |
|  | 9. Participants do not realize the question has changed (EQ-5D-5L) | 8%  (n = 1) | No | Use formatting to differentiate one screen from the next. For example:  • make “mobility” one color and “selfcare” another  • make “mobility” and “self-care” larger so it is more obvious when they change |
| BPDAI pruritus tool | 10. Participants do not realize the question has changed  (BPDAI Pruritus) | 25%  (n = 3) | Yes | Use formatting to highlight the part of the question that is changing. For example, make “the last 24 hours” and “the past week” a larger font, bolded, underlined, and/or italicized.  Progress bars could be a suitable design element to include here. |
| Error message | 11. Error message is unclear | 64%  (n = 7)* | Yes | Change the wording of the error message to note what is wrong and what the user must do next: “You have not selected an answer. Please select an answer and click “next” to proceed.” Also make font size larger to be easier to read |
|  | 12. Error message disappears too quickly | 45%  (n = 5)* | No | Have the error message remain on the screen permanently until the user corrects the issue. |
| Smartphone login | 13. Participants struggle to swipe to unlock the  smartphone | 33%  (n = 4) | Yes | If possible, have the “swipe to unlock” message appear as soon as the screen lights up (not only after a user taps the screen). Also make the font 2–3 times as large, and have it stay on the screen permanently. We also suggest developing a “smartphone 101” training to cover skills like unlocking the phone. This is discussed further below. |
|  | 14. Participants do not know where to enter the  password | 8%  (n = 1) | Yes | Provide instructions at the top of the log-in screen saying, “Tap the white bar below and enter your password” |
|  | 15. Participants struggle to find the “log in” button | 25%  (n = 3) | No | Either:  • Move the log in button to the bottom of the screen, where users look by default  Or  • Visually differentiate the log in button so it stands out (eg, bright green color instead of blue) |
| Smartphone training | 16. Participants  struggle to  navigate the  keyboard | 42%  (n = 5) | No | Bold and/or underline “tap on the box below” in the instructional text. We also suggest changing the wording to “tap on the **bar** below,” per the feedback in issue #1. |
|  | 17. Participants are  confused by  ambiguous mock  questions | 8%  (n = 1) | No | As with issue #3, add specificity to the question. For example, “How satisfied are you **with your itch** today” or “How happy are you right now?” |
|  | 18. Participants do  not know how to  close the training  module | 8%  (n = 1) | No | Like with issue #6, replace the existing question and answers with:  “You have completed the training module. Would you like to  (A) Continue or B) Re-do the training module” |
| OCS medication usage e-diary | 19. “Next” button is  hidden by numeric keyboard | 67%  (n = 8) | Yes | Ensure the “next” button is always visible; it should be visible below the keyboard. If that is not possible from a design perspective, include instructions on how to make it appear, eg, “To proceed, tap the  triangle in the lower right corner, then tap ‘next’.” |
|  | 20. Participants try to input unit of measurement | 33%  (n = 4) | Yes | Populate “mg” to the right of the text bar |
|  | 21. Participants click the checkbox unnecessarily | 33%  (n = 4) | Yes | Ideally: Before users enter OCS dosage, present a screen asking, “Did you take any oral corticosteroid medication over the past 24 hours?” If the user selects “yes”, have them enter their dosage. Remove the check box from the OCS dosage screen.  Alternatively: Remove the check box from the OCS dosage screen. Add an instructional sentence (perhaps under the text box), “If you did not take any oral corticosteroid in the past 24 hours, enter ‘0’.” |
|  | 22. Participants do not mark the checkbox when  indicating they did not take OCS | 25%  (n = 3) | Yes* | See previous solution |
|  | 23. Participants struggle to open the numeric  keyboard | 8%  (n = 1) | No | Adjust the instructions to direct the user to tap the box below. For example: “Tap the box below to enter the dose of …” |
| Smartphone error message | 24. Error message  disappears too  quickly | 36%  (n = 4)* | No | Have the error message remain on the screen permanently until the user corrects the issue. |
|  | 25. Error message  phrasing is  unclear | 9%  (n = 1)* | No | Change the wording of the error message to note what is wrong and what the user must do next: “You have not selected an answer. Please select an answer and click ‘next’ to proceed.” |

*Only 11 participants were tested on the error messages, as this was not added to the interview guide until after the first usability interview was conducted.

#### Supplementary Table 6.2: Insights and general principles regarding usability, training, and compliance

| **Function** | **Recommendation** | **Clarification** |
| --- | --- | --- |
| Usability | Ensure large font size across all screens | Particularly important for populations where disease or treatment impacts eyesight (eg, corticosteroids) |
|  | Accommodate for limited hand dexterity | Avoid complex navigation  Select bright, lightweight devices  Include large and well-spaced buttons (avoiding scales that require users to tap very accurately), or provide a stylus |
|  | Provide comprehensive and specific instructions | Step-by-step instructions  Substantive content in place of mock questions  Ensure messages stay visible and do not disappear after a few seconds |
| Training/compliance | Ensure a live trainer is available, and provide supplemental training resources | Resources could include a hard-copy cheat sheet, a 24-7 help line, online chat, or smartphone 101 training |
|  | Encourage users to establish a routine, and provide electronic reminders | Reminders should appear at a regular time, on personal devices, make an audible sound, and repeat until the task is completed |

### **Section 7: Likelihood of ePROM device use (full dataset)**

Most participants (n = 11; 92%) expressed willingness to use the devices, with only one French participant with BP saying that they would refuse to use either of the eCOA devices, with a preference for pen-and-paper questionnaires. Two participants (17%) thought the devices would be preferable to paper questionnaires. One (8%) noted that electronic versions are faster to complete, and the other (8%) thought patients would appreciate that they and their doctors could monitor symptoms in real time.

Participants did not have a strong preference for one device over the other, but when prompted, responded as follows:

| **Preference** | **% (n)** | **Stated reasons** |
| --- | --- | --- |
| Tablet | 50% (6) | - Larger screen is easier to see and read (n = 6; 50%) - Harder to lose (n = 1; 8%) |
| Smartphone | 33% (4) | - Smaller size, so easier to carry and hold (n = 2; 17%) |
| No preference/depends | 17% (2) | - Prefer tablet at sites and smartphone at home (n = 1; 8%) - Prefer tablet for tasks where more information must be presented on the screen (n = 1; 8%) |

#### 7.1 Tablet

Participants were asked how likely they would be to complete the tablet tasks, with the understanding that these would be completed during site visits. Most (n = 7; 58%) indicated nothing would prevent them from completing the assigned tasks.

| **Barriers to tablet use** | **Mentions** |
| --- | --- |
| Nothing | 58% (n = 7) |
| Attending site visits | 25% (n = 3) |
| Difficulty making site visits due to scheduling constraints | 8% (n = 1) |
| Difficulty physically travelling to site due to mobility issues | 17% (n = 2) |
| Remembering how to complete the tasks | 17% (n = 2) |
| Sickness/depression | 8% (n = 1) |

### **Section 8: Training and support (full dataset)**

At the end of each interview, participants were asked to evaluate how well prepared they felt to complete the tasks. Overall, participants were pleased with the training modules offered on both the tablet and the smartphone, describing them as sufficient, straightforward, and helpful. One participant (8%) emphasized the value of having an in-person trainer to demonstrate the tasks and provide support as they attempted the task themselves, ensuring complete understanding. Overall recommendations included allowing participants the opportunity to take the training again at any point in their trial journey and adding a “smartphone 101” training for participants with less technology fluency. Additional support resources are covered in **Suppl. Section 6, Table 2**.
